# Predicting blood pressure from physiological index data using the SVR algorithm

**DOI:** 10.1186/s12859-019-2667-y

**Published:** 2019-02-28

**Authors:** Bing Zhang, Huihui Ren, Guoyan Huang, Yongqiang Cheng, Changzhen Hu

**Affiliations:** 1 School of Information Science and Engineering, Yanshan University, Hebei Avenue, Qinhuangdao, 066004 China; 2 The Key Laboratory for Computer Virtual Technology and System Integration of Hebei Province, Hebei Avenue, Qinhuangdao, 066004 China; 3 Department of Computer Science and Technology, University of Hull, Hull, HU6 7RX UK; 4 Beijing Key Laboratory of Software Security Engineering Technique, Beijing Institute of Technology, Beijing, 100081 China

**Keywords:** Physiological index data, SVR, Blood pressure prediction

## Abstract

**Background:**

Blood pressure diseases have increasingly been identified as among the main factors threatening human health. How to accurately and conveniently measure blood pressure is the key to the implementation of effective prevention and control measures for blood pressure diseases. Traditional blood pressure measurement methods exhibit many inherent disadvantages, for example, the time needed for each measurement is difficult to determine, continuous measurement causes discomfort, and the measurement process is relatively cumbersome. Wearable devices that enable continuous measurement of blood pressure provide new opportunities and hopes. Although machine learning methods for blood pressure prediction have been studied, the accuracy of the results does not satisfy the needs of practical applications.

**Results:**

This paper proposes an efficient blood pressure prediction method based on the support vector machine regression (SVR) algorithm to solve the key gap between the need for continuous measurement for prophylaxis and the lack of an effective method for continuous measurement. The results of the algorithm were compared with those obtained from two classical machine learning algorithms, i.e., linear regression (LinearR), back propagation neural network (BP), with respect to six evaluation indexes (accuracy, pass rate, mean absolute percentage error (MAPE), mean absolute error (MAE), R-squared coefficient of determination (*R*^2^) and Spearman’s rank correlation coefficient). The experimental results showed that the SVR model can accurately and effectively predict blood pressure.

**Conclusion:**

The multi-feature joint training and predicting techniques in machine learning can potentially complement and greatly improve the accuracy of traditional blood pressure measurement, resulting in better disease classification and more accurate clinical judgements.

## Background

Blood pressure is an important physiological parameter that reflects the state of the cardiovascular system and is playing an increasingly important role in clinical work. Regular monitoring of blood pressure is conducive to the early detection and diagnosis of various types of blood pressure disorders to ensure timely treatment and prevention. Therefore, the development of medical devices to rapidly and accurately measure blood pressure is of great significance.

Two methods are used clinically to measure blood pressure, i.e., the direct approach and indirect approach. On the one hand, in the direct method, the blood pressure measuring system is directed at blood vessels, even those in the region of the heart. The direct method has the characteristics of low signal distortion, but the measurement is complex and has corresponding health safety risks. On the other hand, the indirect measurement has become increasingly accurate and is widely used in clinical practice. Auscultation and oscillometry are commonly used indirect and intermittent blood pressure measurement methods. Auscultation measures systolic blood pressure (Ps) and diastolic blood pressure (Pd) through the Korotkoff sound, of which the reading suffers from subjective effects, error, poor repeatability and susceptibility to noise interference. The oscillographic approach has better reliability and accuracy. The oscillographic method can also measure mean blood pressure [1]. Oscillographic approaches include the amplitude coefficient method [2, 3] and the waveform characteristic method [4, 5]. Improved oscillographic methods include the variable coefficient method [6], the difference ratio method, the inflection point method and the combination method [7]. Oscillographic approaches are based on changes in the characteristics of the waveform data in the cycles of a pulse wave. In the amplitude coefficient method and its improved version, the accuracy of the amplitude coefficient is difficult to guarantee due to the existence of individual differences. For the waveform feature method and its improvement version, the pulse intensity and pulse changes in a cycle vary among users due to differences between individuals, which may cause the characteristic data fail to satisfy the requirements of the blood pressure prediction method [8].

In addition, the application of intelligent devices in the medical field has gained increasing attention [9], and the use of portable intelligent devices instead of medical equipment to detect human vital signs, such as heart rate (HR) [9–16], respiratory rate [11], blood oxygen saturation level (SpO2) [11], and pulse rate [13] has become more common. Considerable medical data with great research value have been generated in the process. Analysing the data through data mining, machine learning and other analytical techniques is an important current trend. Furthermore, this trend has inspired us to use machine learning algorithms to investigate the hidden knowledge in the substantial amount of medical data collected by intelligent devices. These data can assist the medical staff to perform disease diagnosis, especially blood pressure disease [14, 17] prediction and prevention. We can identify and analyse the complex mapping relationships between blood pressure and human physiological indexes by machine learning mechanisms to establish efficient blood pressure prediction models. The multi-feature joint training and predicting techniques in machine learning can potentially complement traditional blood pressure measurement and will greatly improve the accuracy, resulting in better diseases classification and more accurate clinical judgements.

Many studies have applied machine learning algorithms to blood pressure prediction. Wu et al. [15] analysed the association between blood pressure and personal circumstances, including body mass index (BMI), age, exercise level, drinking and smoking, by means of artificial neural networks. Golino et al. [18] used categorical trees to predict blood pressure variance trends based on BMI, waist circumference (WC), hip circumference (HC), and waist–hip ratio (WHR). Both studies focused on the correlations between human body health data and blood pressure to estimate the readings. Some other physiological parameters, such as electrocardiograph (ECG), photoplethysmography (PPG), and heart sound signals, have a more direct relation with blood pressure. In [19], two neural network algorithms were used to predict Ps by correlated factors (gender, serum cholesterol, fasting blood sugar and ECG signal). The paper [20] dealt with the accurate evaluation of the blood pressure by an Artificial Neural Network and the PPG signal. The authors in [21] used the ensemble neural network algorithm to model the relationship between PPG and blood pressure. Moseley et al. [22] used cardiovascular reactivity and recovery to predict long-term blood pressure and HR. Cardiovascular reactivity and recovery are laboratory stress-induced cardiovascular changes that can be used to predict trends in blood pressure and HR over the next three to ten years. Ghosh et al. [23] combined ECG with PPG characteristic data signals and calculated the pulse wave transmission time (PTT), which has been used in LinearR models to predict blood pressure. Peng et al. [24] used the heartbeat signal characteristics to establish a support vector machine (SVR) regression model for continuous and cuffless blood pressure measurement, but the predictive accuracy was not high. Kurylyak et al. [25] used a PPG signal to establish a neural network model to predict blood pressure continuously. However, the accuracy was not ideal when the model was evaluated with respect to the absolute error and relative error index. Hsieh et al. [26] used a dynamic PTT to establish a LinearR model for noninvasive blood pressure prediction using the MAE and *R*^2^ to evaluate model. However, the Ps was not ideal. He et al. [27] attempted to use a random forest model to predict blood pressure and evaluated the model in terms of accuracy and MAE. However, the MAE was large, and the accuracy of the systolic pressure prediction model was low.

In this paper, the SVR model using radial basis function (RBF) is applied to mine the relationship between human physiological data and blood pressure to establish a Ps and Pd model for effective and accurate blood pressure prediction. The main physiological indexes of the human body include PTT, HR, PPG. The blood pressure measurements include Ps and Pd, which are measured by existing electronic blood pressure gauges. The SVR model based on RBF can handle the case when the relation between target labels and attributes is nonlinear, which performs better than other models. In this paper, parameter optimization of the SVR training model was performed based on 10-fold cross-validation, and the optimal parameters C and g were found, respectively, in the Ps and Pd SVR prediction models. Then, the SVR prediction models were established based on the optimal parameters. The training set and test set were randomly divided with a ration of 4:1. The training set was used to train the SVR model, and the test set was used to assess the model predictions. Finally, the accuracy, pass rate, MAPE, MAE [28], *R*^2^ [29] and Spearman’s correlation coefficient [30, 31] were used to evaluate the predictive ability of the model to verify the feasibility and efficiency of the SVR model in the prediction of blood pressure. Finally, the prediction results of two other classical machine learning algorithms, namely, LinearR, BP were compared. The results show that the prediction of blood pressure by the SVR model is the best.

1. The optimal parameters C and g of the SVR models are found based on 10-fold cross-validation, based on which the SVR model is established.
2. The performance of the SVR model is evaluated in terms of six evaluation indexes.
3. Based on SVR prediction method proposed, it becomes promising to realize a highly efficient and real-time continuous blood pressure monitoring.

The rest of this paper is organized as follows. The methods section describes the data collection and processing, some important features, the six evaluation indexes, and the principles of the SVR model and prediction process. The experimental data and results are given in results section. The discussion and conclusion sections, respectively, state the limitations of the study and the main conclusions.

## Methods

### Data collection and processing

Eighteen participants (12 males, 6 females) displayed in Table 1 were recruited for the study. Participants were included in the study if they were apparently healthy males or females between 18 and 50 years old, non-smokers, asymptomatic, with less than or equal to one coronary artery disease risk factor, no family history of myocardial infarction, obesity, hypertension, dyslipidemia, or heart disease, no history of cardiovascular, renal, hepatic, thyroid disease, and no history of physical inability to exercise. Participants were required to adhere to the following pre-measurement guidelines:

1. Refrain from caffeine for at least 4 h.

**Table 1:** Participant characteristics

Characteristic	Overall mean ±SD	Mean ±SD	Mean ±SD
	*n*=18 all	*n*=12 males	*n*=6 females
Age (years)	29 ±9	30 ±9	28 ±10
Body mass (kg)	76.9 ±16.5	85.0 ±13.8	60.7 ±5.5
Height (cm)	174.0 ±9.0	178.0 ±5.7	165.9 ±9.4
BMI (kg/ *m*^2^)	25.3 ±4.5	26.9 ±4.7	22.1 ±1.7
Body fat (%)	18.7 ±5.2	18.2 ±5.5	19.5 ±5.9

SD = Standard Deviation
2. Refrain from any unhabituated strenuous exercise sessions at least 24 h prior to each testing day.
3. Refrain from alcohol consumption for at least 24 h prior to testing.
4. Maintain normal hydration by drinking to thirst.
5. Consume a light carbohydrate meal no less than 2 h prior to testing.

**Fig. 1:**
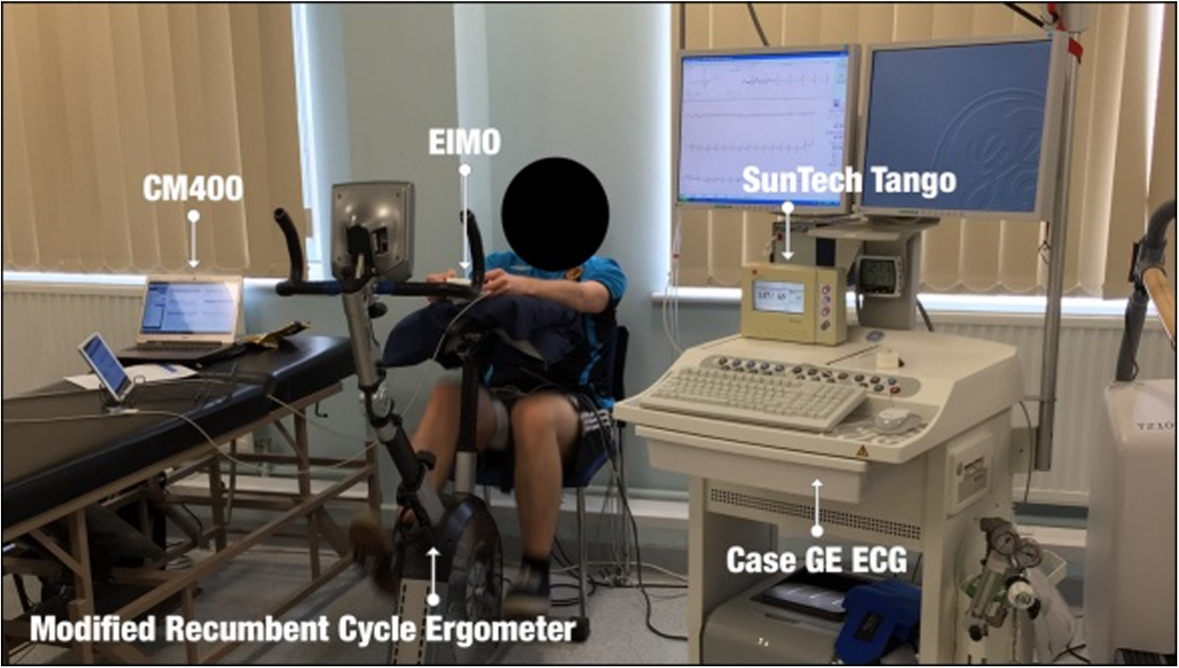
The setup of the data collection

Following familiarization, participants completed three rest and exercise sessions incorporating concurrent Ps and Pd measurements from both a cuffless blood pressure measurement device (EIMO) and the criterion system (SunTech Tango automated monitor). Each session included a 30 min rest period during which six blood pressure measurements were obtained. Participants then completed three 15 min exercise periods at 25, 50, and 75 w for a total of 45 min. ECG and PPG were measured continuously by a patient monitor (CM400), a 6-lead ECG (Case GE ECG system) and the EIMO device during the rest and exercise phases. Twelve blood pressure measurements were taken by the SunTech Tango system at 2 min intervals during the exercise period, with a measurement taken after 2 min of recovery cycling. The setup of the data collection is shown in Fig. 1.

**Fig. 2:**
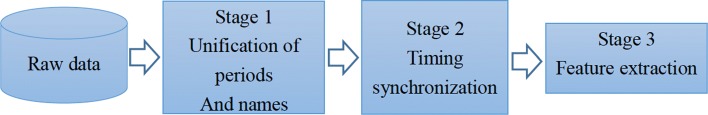
Data analysis flow

Data analysis and processing was conducted in 3 stages to more easily rationalize the analysis steps. A workflow of the analysis process is shown in Fig. 2. After the first two steps, the feature data set and the blood pressure data set were matched by their time stamps, and two features (PTT, HR and SpO2) were extracted in the third step. PTT was computed using windowed correlation between the ECG and PPG signals, and HR was extracted from the ECG signal and SpO2 was extracted from the PPG signals.

The final experimental data include 10 characteristics: 6-lead ECG (I, II, III, aVF, aVL, and aVR), PPG features from the Case GE system, PTT, HR and SpO2 obtained via feature extraction. The feature selection process was designed in consideration of two aspects, namely, Spearman’s correlation [31] between features and a mutual information (MI) [32] coefficient between each feature and the target value. The range of Spearman’s correlation coefficient is (-1, 1). The greater the absolute value is, the stronger the correlation is, and the sign of the value indicates the relevant direction. MI measures the dependency between the variables of the feature and target. MI is equal to zero if and only if two variables are independent, and higher values indicate stronger dependence. We conducted a reasonable feature selection process to obtain more effective blood pressure predictions; the process and basis are presented in the feature analysis and selection subsection of the results section. The following contents briefly introduce five important features: PTT, HR, PPG, aVF, and *SpO*2.

### Attribute description

Among the wide range of human physiological indicators that can be collected by intelligent devices, the main indicators impacting human blood pressure are PTT, HR, PPG, aVF and *SpO*2. PTT is calculated based mainly on the pulse wave mapped from internal human heart activity state to determine the blood pressure of the human body. HR is a vital sign that has been well described in the normal population and in various pathological states. HR and blood pressure are closely correlated, and hypertensive patients have higher resting HR than normotensive patients [33]. Blood pressure can be measured continuously based on ECG and *SpO*2 [34] or based on ECG and PPG signals [23]. PPG, aVF and *SpO*2 are common important physiological parameters that affect human blood pressure.

### Evaluation index

#### Accuracy

For regression model of blood pressure prediction, this paper defines the accuracy evaluation index under different error ranges, as shown in formula (1), where x represents the number of correct predictions and y represents the total number of model predictions. If the difference between the predicted and actual values is within a certain range (the acceptable error according to the ANSI/AAMI SP10-1992 standard is ±5 mmHg), the prediction results are considered to be correct.

1 $$ accuracy = \frac{x}{y}  $$

#### Pass rate

In general, the relative error, defined in formula (2), where pre_val and real_val represent the predicted and actual values respectively, can accurately reflect the credibility of a measurement. In this paper, the pass rate of the blood pressure prediction model is required to be in accordance with different relative errors (±0.03, ±0.05, ±0.07, ±0.1). That is to say, we first calculate the relative error of the predicted blood pressure and then obtain the ratio of the number of predicted values that satisfy different relative error ranges to the total number of test samples.

2 $$ re\_err = \frac{real\_val-pre\_val}{real\_val}  $$

#### MAE

The MAE is the average of the absolute values of the deviation between the predicted and actual values. Since the deviation is absolute, there is no positive and negative offset. MAE is not sensitive to the effect of anomalies, but it can reflect the actual situation of the prediction error. MAE is calculated by formula (3), where n is the number of samples, and *y*_*i*_ and $\hat {y_{i}}$ represent the actual blood pressure and that predicted by the model for the i-th sample.

3 $$ MAE = \frac{1}{n}\sum\limits_{i=1}^{n}\left|\left(y_{i}-\hat{y_{i}} \right) \right|  $$

#### MAPE

To show the range of model prediction error overall, the MAPE is defined as the mean of the absolute value of the relative error. In the formula (4), n represents the number of samples, and re_err represents the relative error, as shown in formula (2).

4 $$ MAPE = \frac{1}{n}\sum_{i=1}^{n}|(re\_err)|  $$

#### *R*^2^

The *R*^2^ can be used to determine whether the features describe the target value. In this paper, the *R*^2^ refers to the comprehensive effect of all the characteristics on the blood pressure prediction, rather than the effect of each individual characteristic on blood pressure. *R*^2^ ranges from 0 to 1. The closer the value is 1, the stronger the degree of the interpretation. *R*^2^ is calculated as shown in formula (5), where n represents the number of samples, *y*_*i*_ represents the actual blood pressure value of the i-th sample, $\hat {y_{i}}$ represents the predicted value, and $\bar {y}$ represents the average of all predicted values, as shown in formula (6).

5 $$\begin{array}{@{}rcl@{}} R^{2}\left(y,\hat{y}\right) =1- \frac{\sum_{i=1}^{n}{\left(y_{i}-\hat{y_{i}}\right)}^{2}}{\sum_{i=1}^{n}{\left(y_{i}-\bar{y}\right)}^{2}} \end{array} $$

6 $$\begin{array}{@{}rcl@{}} \bar{y} = \frac{1}{n}\sum_{i=1}^{n}y_{i} \end{array} $$

#### Spearman’s rank correlation coefficient

Spearman’s rank correlation coefficient, which is a nonparametric statistical method, does not require the distributions of the original variables to be known. Spearman’s rank correlation coefficient is a measure of the degree of correlation between hierarchical variables. It is also called the rank correlation coefficient and takes between -1 and 1. The greater the absolute value is, the stronger the correlation is, and the sign of the value indicates the relevant direction. Spearman’s rank correlation coefficient is calculated by formula (7), where *R*_*i*_ represents the rank of *y*_*i*_, *Q*_*i*_ represents the rank of $\hat {y_{i}}$, *y*_*i*_ and $\hat {y_{i}}$, respectively, represent the actual blood pressure value and the value predicted by the model for the i-th sample. In this paper, the P-value is also calculated to test the null hypothesis of no correlation against the alternative hypothesis that there is a nonzero correlation. Small *P*-value, for example, less than 0.05, indicate that the correlation is significantly different from zero.

7 $$ r = 1-\frac{6\sum_{i=1}^{n}(R_{i}-Q_{i})^{2}}{n\left(n^{2}-1\right)}  $$

### Comparison model

#### LinearR

In LinearR target value is expected to be a linear combination of the input variables. In mathematical notion as shown in formula (8) where we designate the vector w = (*w*_1_,..., *w*_*p*_) as parameters of features and *w*_0_ as intercept, if $\hat {y}$ is the predicted value.

8 $$ \hat{y}(w, x) = w_{0} + w_{1} x_{1} +... + w_{p} x_{p}  $$

LinearR fits a linear model with coefficients w = (*w*_1_,..., *w*_*p*_) to minimize the residual sum of squares between the observed responses in the data set and the responses predicted by the linear approximation. Mathematically it solves a problem of the form as shown in formula (9):

9 $$ \min_{w} {|| X_{w} - y||_{2}}^{2}  $$

Considering that coefficient estimates for Ordinary Least Squares rely on the independence of the model terms, we have mapped all features into a uniform distribution to normalize them. After training, the parameters of Ps and Pd models were obtained as following Table 2. *w*_1_- *w*_3_ represents the parameters of three features (PTT, HR, PPG), *w*_0_ as intercept.

**Table 2:** The parameters of LinearR

Model	Ps	Pd
*w*_1_(PTT)	-2.6707	0.2456
*w*_2_(HR)	3.6058	-1.2704
*w*_3_(PPG)	-0.3166	0.2623
*w*_0_	127.8482	64.6456

#### BP model

**Fig. 3:**
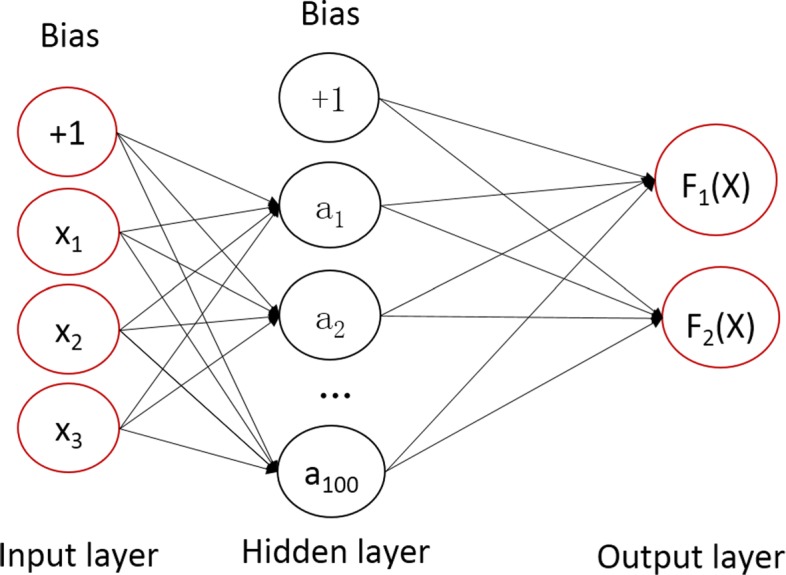
The network structure of BP model

BP is a supervised learning algorithm that learns a function f (·): *R*^*m*^ → *R*^*o*^ by training on a data set, where m is the number of dimensions for input and o is the number of dimensions for output. Given a set of features X = *x*_1_, *x*_2_,..., *x*_*m*_ and a target y, it can learn a non-linear function approximator for either classification or regression. Between the input and the output layer, there can be one or more non-linear layers, called hidden layers. The structure of a three-layer neural network model designed in this paper is shown in the following Fig. 3.

The leftmost layer, known as the input layer, consists of a set of neurons {*x*_*i*_ | *x*_1_, *x*_2_,...,*x*_3_} representing the input features (PTT, HR, PPG). Each neuron in the hidden layer (including 100 neurons) transforms the values from the previous layer with a weighted linear summation *w*_1_*x*_1_+*w*_2_*vx*_2_+*w*_3_*x*_3_, followed by a non-linear activation function the hyperbolic tan function. The output layer receives the values from the last hidden layer and transforms them into output values. The two neurons in the output layer represent the predicted value of Ps and Pd respectively.

### SVR model

A support vector machine consists of a series of supervised machine learning algorithms that is used to solve classification, regression, and abnormality detection problems. A support vector machine contains a variety of models that can be classified as linear separable support vector machines, linear support vector machines and nonlinear support vector machines. The basic idea of linear separable and linear support vector machines is to construct the linear classifier that has the largest distance in the feature space and can address linearly separable data objects. For complex nonlinear classification problems, the nonlinear support vector machine is adopted. The nonlinear support vector machine transforms linearly non-separable problems in low-dimensional space into linearly separable problems in high-dimensional space via a kernel function. A subset of support vectors are used in the training set to represent the decision boundary. Due to the nature of nonlinear regression using complex characteristics of human physiological index data to predict blood pressure, we envisioned the feasibility of using the nonlinear support vector machine to predict human blood pressure.

#### Kernel function selection

Kernel functions can simulate the projection of the initial data in a feature space with higher dimension where the data are considered as linearly separable, so that kernel functions can help to establish nonlinear support vector machines models. At present, there are three main classes of kernel functions [35]: polynomial kernel functions, RBF and sigmoid kernel function as shown in formula (10)–(12). Different kernel functions will produce different algorithms, and the data will be mapped onto different feature spaces.

10 $$\begin{array}{*{20}l} k\left(x,x^{\prime}\right)&=\left(\gamma \left\langle x, x^{\prime} \right\rangle + r \right)^{d}  \end{array} $$

11 $$\begin{array}{*{20}l} k\left(x,x^{\prime}\right)&=\exp\left(-\gamma \left\|x-x^{\prime} \right\|^{2} \right)  \end{array} $$

12 $$\begin{array}{*{20}l} k\left(x,x^{\prime}\right)&=\tanh \left(\gamma \left\langle x,x^{\prime} \right\rangle + r \right)  \end{array} $$

The RBF kernel is adopted in the SVR algorithm by mapping the original feature space X = (PPG, PTT, HR) onto the new feature space X’ = (*x*_1_, *x*_2_, *x*_3_,... *x*_*n*_). The finite set of blood pressure indicator data can be expressed by a linear regression formula in the new feature space to establish a nonlinear mapping model between human physiological index data and blood pressure. In addition, the RBF kernel has the advantage of fewer parameters than the polynomial kernel, and the number of parameters directly affects the complexity of model selection. Therefore, an SVR model based on the RBF kernel function has low complexity. This paper also attempts to establish an SVR model based on a polynomial kernel function and a sigmoid kernel function, but the polynomial SVR model need a too long parameter optimization process to convergence and the sigmoid SVM model has fast parameter optimization but the predictions are poor.

Two parameters, namely, C and gamma, are involved in the model training process when using the RBF kernel function. Parameter C is a common parameter for all kernel functions used in support vector machines and can be used to balance the classification error in the training set and the smoothness of the decision plane [36]. A smaller C makes the decision surface smoother, whereas a larger C allows the model more freedom to use more samples as support vectors so that all the training samples can be accurately classified or predicted. Gamma is used to adjust the influence of a training sample. The larger the gamma value is, the smaller the influence of the training sample. Thus, proper values of parameters C and gamma are critical to the performance of the SVR model.

#### Description of SVR

The basic idea of SVR is to map the input space onto a high-dimensional feature space by nonlinear mapping and then linearly solve nonlinear problems [37]. In this paper, the vector in input space is x = (PPG, PTT, HR), assuming that the nonlinear model is:

13 $$ \hat{f}(x,\omega)=\omega\phi(x)+b  $$

Assuming that all training samples can be fitted by a linear function without error under, the relaxation factor is introduced to handle data that cannot be estimated by the function at a specified precision threshold. In (13), *ω* and b can be obtained by solving the following optimization problem:

14 $$ {min}_{\omega,b,\xi,\xi^{*}}\frac{1}{2}\omega^{T}\omega+C\sum\limits_{i=1}^{n}\left(\xi_{i}+\xi_{i}^{*}\right)  $$

subject to:

15 $$ \begin{aligned} y_{i}-\left[\omega^{T}\phi(x_{i})+b\right]&\leq\varepsilon+\xi_{i} &\\ \left[\omega^{T}\phi(x_{i})+b\right]-y_{i}&\leq\varepsilon+\xi_{i}^{*} &\\ \xi_{i},\xi_{i}^{*} &\geq0,i=1,...n & \end{aligned}  $$

Using the dual principle, the Lagrangian multiplier and the kernel function, formula (14) is transformed into the following dual optimization problem:

16 $$ {\begin{aligned} {min}_{\alpha,\alpha^{*}}\frac{1}{2}\sum\limits_{i=1}^{n} \sum\limits_{j=1}^{m} \left(\alpha_{i}-\alpha_{i}^{*}\right) \left(\alpha_{j}-\alpha_{j}^{*}\right) k(x_{i},x_{j}) &+ \varepsilon\sum\limits_{i=1}^{n} \left(\alpha_{i}+\alpha_{i}^{*}\right)\\ & - y_{i} \sum\limits_{i=1}^{n}\left(\alpha-\alpha^{*}\right) \end{aligned}}  $$

subject to:

17 $$ \sum\limits_{i=1}^{n}\left(\alpha_{i}+\alpha_{i}^{*}=0 \right)\qquad {\alpha_{i},\alpha_{i}^{*}\in[0,c],i=1,2,...,n}  $$

where *α*_*i*_, $\alpha _{i}^{*}$ is the Lagrangian multiplier, and k (*x*_*i*_, *x*_*j*_) is the kernel function. After solving (16), the regression function (support vector) of formula (13) becomes:

18 $$ f(x)=\sum\limits_{i=1}^{n}\left(\alpha_{i}-\alpha_{i}^{*} \right)K(x_{i},x)+b  $$

#### SVR blood pressure prediction process

Figure 4 shows the detailed prediction process. The process of blood pressure prediction includes 5 steps:

1. After extration and combination of features from the data collected, we have mapped all features to a uniform distribution with values between 0 and 1 to normalize them and divided the data into a training set and test set (4:1 ratio).

**Fig. 4:**
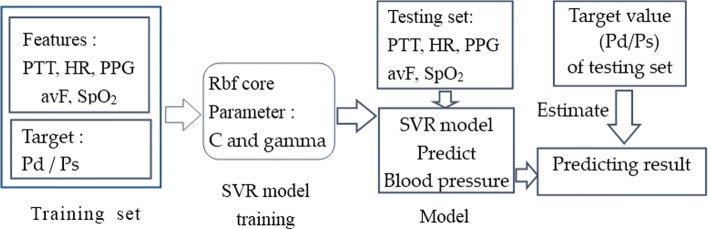
Schematic diagram of diastolic pressure prediction
2. Establish a Pd and Ps SVR model, and initialize the model using the optimal parameters C and g, which were obtained by the optimization function based on 10-fold cross-validation using the training set.
3. Feed the training set, including the feature vector of the human physiological index data and the blood pressure target vector, into the SVR model to train the model. The prediction model is obtained from the training process.
4. Feed the characteristic set of the test set into the trained model to predict the corresponding blood pressures.
5. Compare the predicted blood pressure values with the values measured by medical devices to calculate the deviations and the values of the six evaluation indexes.

## Results

### Data set analysis

**Fig. 5:**
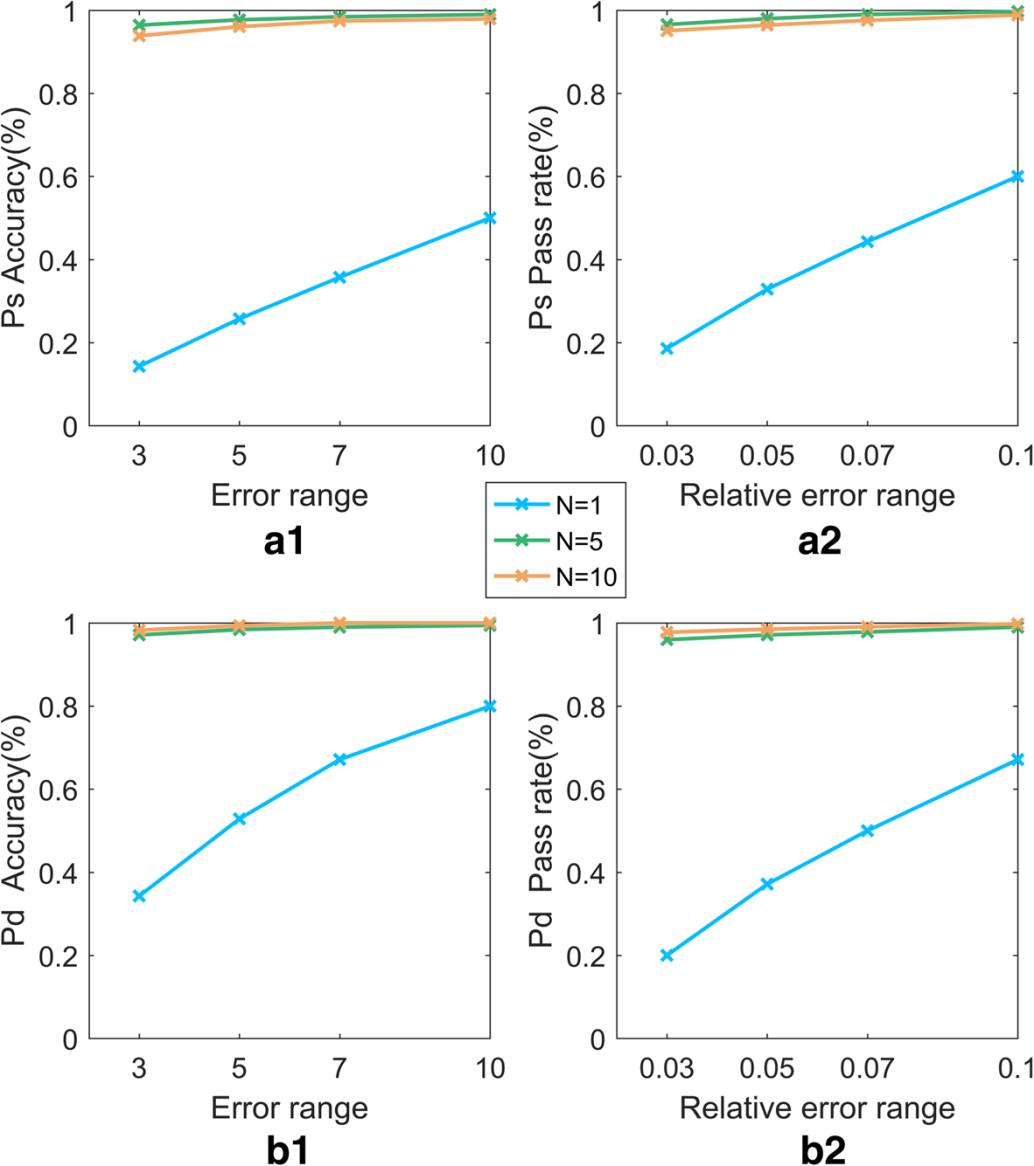
Comparison of the accuracy and pass rate of models with different N. In Fig. 5, (a1) and (a2), respectively, represent the accuracy and pass rate for the models of Ps, while (b1) and (b2) represent those of the models of Pd. The blue, green, and orange lines represent the performance of the models with different 2N windows

**Fig. 6:**
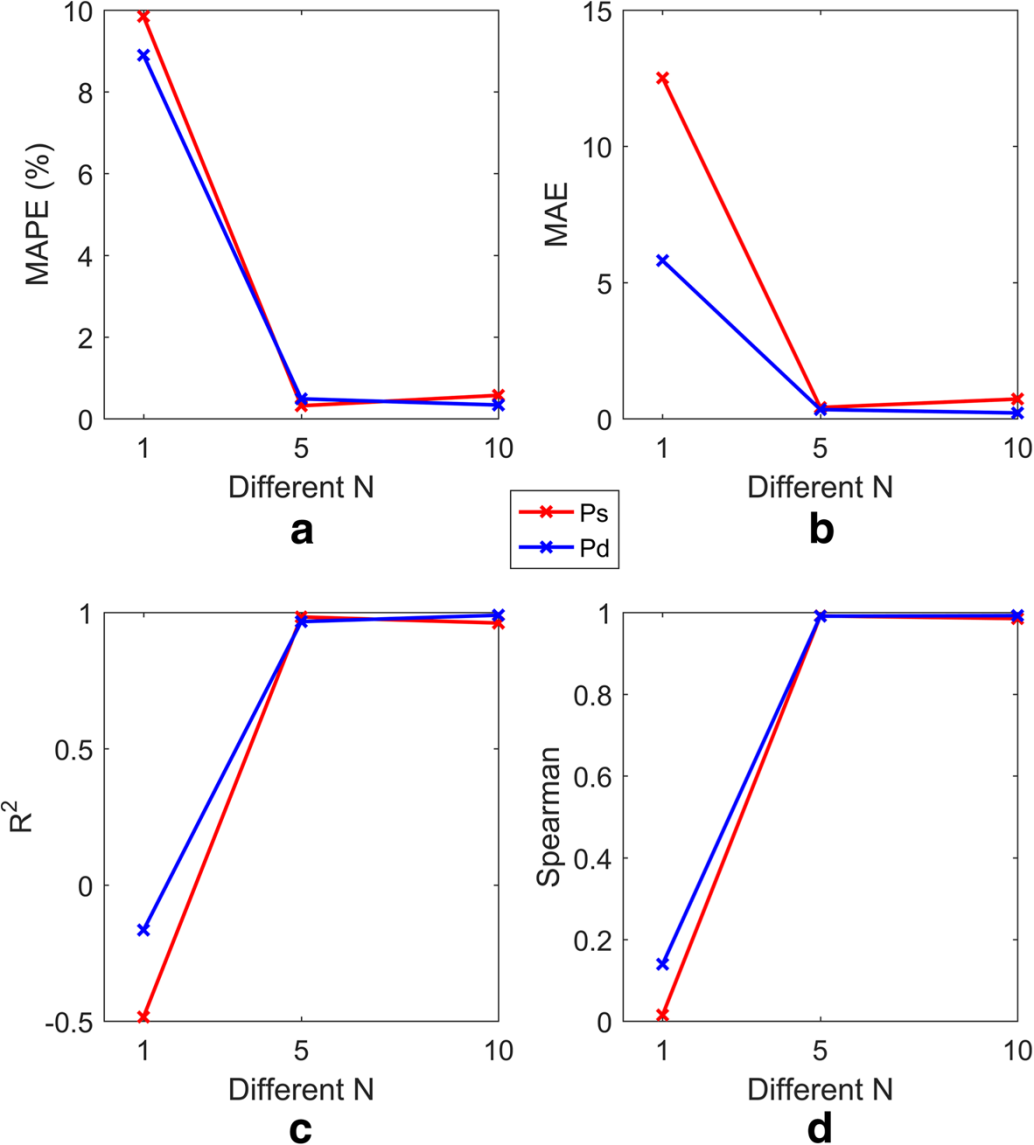
Comparison of four evaluation indexes for models with different N. In Fig. 6, **a**, **b**, **c**, and **d**, respectively, represent the performance of models (including Ps and Pd) with respect to MAPE, MAE, *R*^2^ and Spearman’s correlation for different 2N windows. The red and blue lines represent the Ps and Pd models, respectively

The 15628501 human physiological index data contain the characteristic data of the human body in different states during rest and under different exercise loads. The characteristic items include PTT, HR, PPG, I, II, III, aVF, aVR, aVL, *SpO*2, and BP(including Pd and Ps). The data were sampled at 100 Hz (i.e., in 0.01 second intervals) and saved in a single file. The blood pressure data were collected by an electronic blood pressure instrument and saved in another file. The feature data set and the blood pressure data set were matched by time stamps. Given the short time interval between adjacent feature records, the blood pressure values do not undergo sudden changes; hence, a 2N window is used in the feature records to include N records prior to the exact matched value and N-1 records after the matched time while maintaining the same blood pressure values. The performance of the model is improved by a larger N, but the performance becomes relatively stable or even degrades and time consumption increases at increasingly larger values of N. Therefore, *N*=5 was used in our experiment to include the records 0.05 s before and after the specified time point. When *N*=5, the data set contains 3500 samples. The data sets corresponding to 3 different N values were randomly divided into training sets and test sets in a 4:1 ratio, and three sets of 10-fold cross-validation model prediction experiments were conducted. The three sets of model prediction results are shown in Figs. 5 and 6.

Figures 5 and 6 show the results of the Ps and Pd prediction models with the optimal parameters, in which the parameters (C, gamma) are set to (100, 10) and (100, 10) and the RBF kernel function is used. The algorithm runs on a computer with a Windows 7 64-bit operating system, 4 GB RAM, and an Intel (R) Core (TM) i5-3210 CPU @ 2.50 GHz processor. The simulation environment is MATLAB.

### Feature Analysis and Selection

**Fig. 7:**
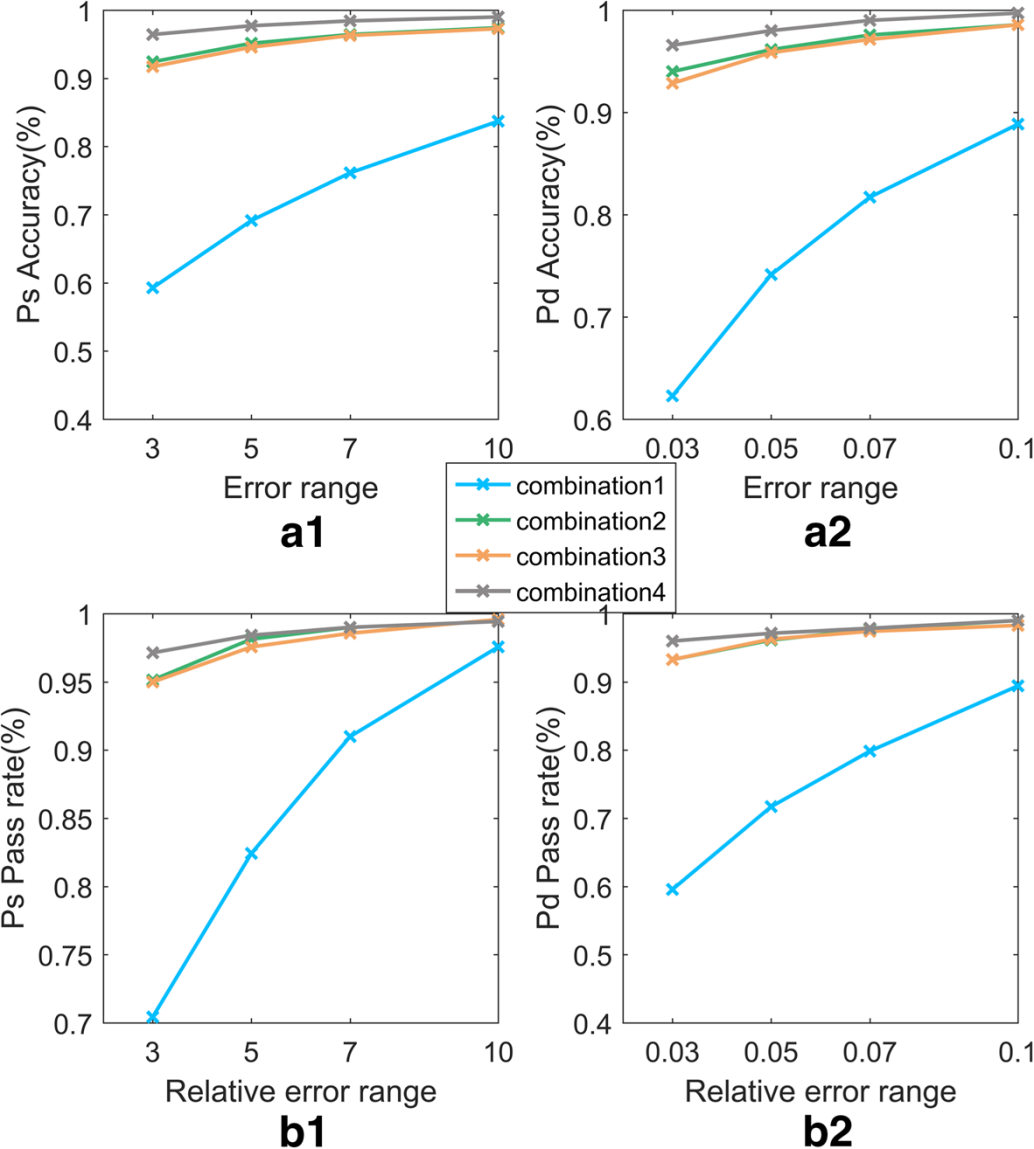
The accuracy and pass rate of the SVR models with 4 different feature combinations. In Fig. 7, (a1) and (a2), respectively, represent the accuracy and pass rate for the models of Ps, whereas (b1) and (b2) represent those for the models Pd. The blue, green, orange and grey lines represent for the performance of the models with the feature combinations shown in Table 5

**Fig. 8:**
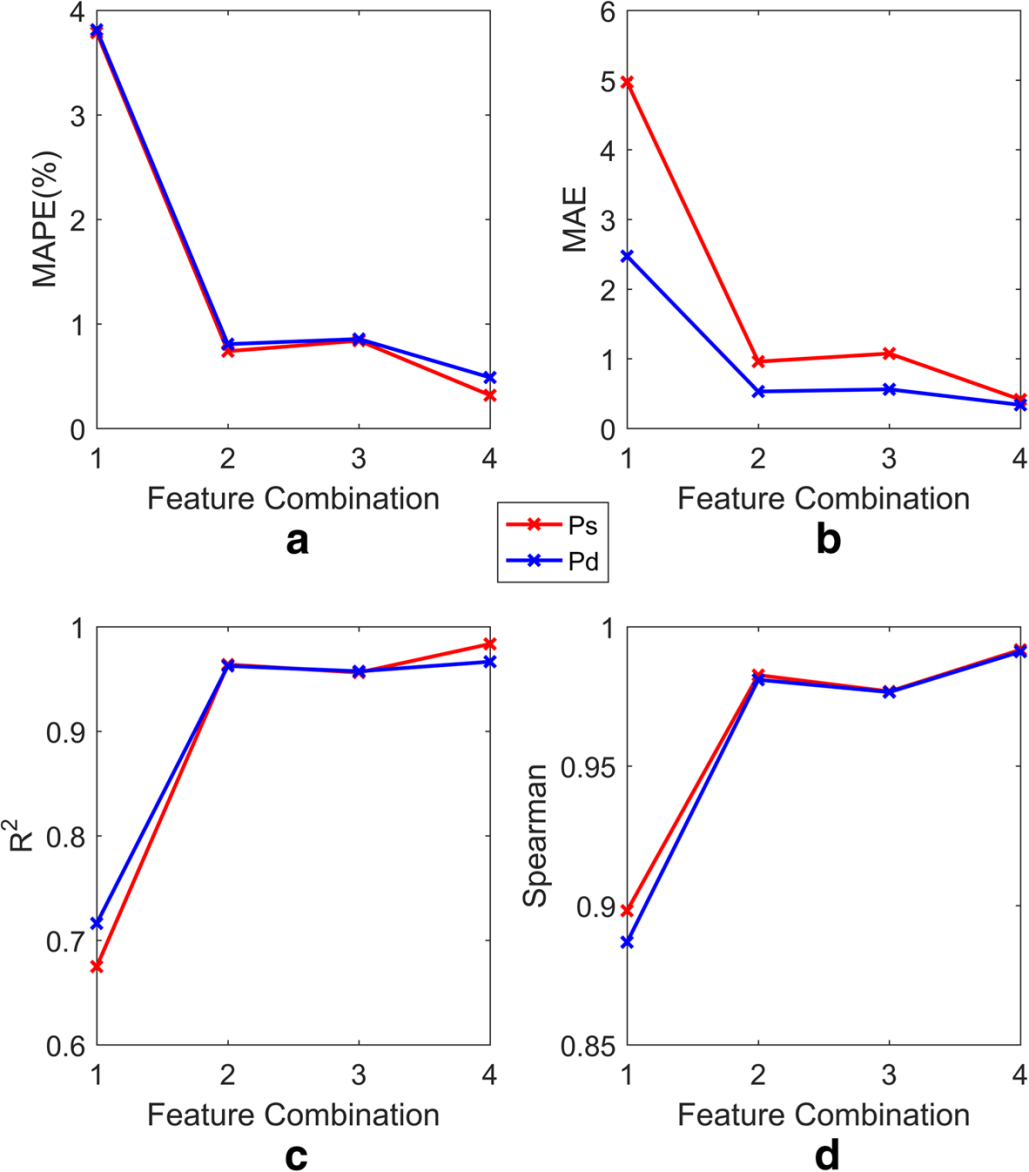
MAPE, MAE, *R*^2^, Spearman’s correlation of SVR models with 4 different feature combinations. In Fig. 8, **a**, **b**, **c**, and **d**, respectively, represent the performance of models (including Ps and Pd) in terms of MAPE, MAE, *R*^2^ and Spearman’s correlation for the feature combinations shown in Table 5. The red and blue lines represent the Ps and Pd models, respectively

Two aspects, namely, the correlation between features and the dependence of each feature on blood pressure, are considered when performing feature selection. The correlation among the 10 features and that between each feature and the target value (Ps, Pd) are analysed based on Spearman’s correlation coefficient and the MI coefficient (the results are shown in Tables 3 and 4). All features are ranked from high to low according to their degree of dependence on blood pressure, which is indicated by MI (the results are shown in Table 4). Tables 3 and 4 show that there is a strong dependence between blood pressure (Ps and Pd) and PPG, PTT, and HR. In addition, there is no substantial difference between the dependence of the other features (I, II, III, aVR, aVL, aVF and *SpO*2) and blood pressure. However, there is a significant correlation among the six features I, II, III, aVR, aVL, and aVF. Considering these two aspects comprehensively, Table 5 lists the combinations of features and the corresponding reasons. The performances of the models with 4 different feature combinations (when *N*=5) are shown in Figs. 7 and 8.

**Table 3:** Correlation analysis of all the features

	I	II	III	aVR	aVL	aVF	PPG	*S* *p* *O*_2_	PTT	HR
I	1	0.3628	0.0346	0.6301	0.4168	0.1747	0.1295	0.0027	0.0930	0.1062
II	0.3628	1	0.9182	0.9518	0.6936	0.9808	0.1127	0.0280	0.1028	0.0271
III	0.0346	0.9182	1	0.7525	0.9223	0.9778	0.0658	0.0289	0.0708	0.0160
aVR	0.6301	0.9518	0.7525	1	0.4396	0.8737	0.1364	0.0242	0.1161	0.0576
aVL	0.4168	0.6939	0.9223	0.4396	1	0.8210	0.0097	0.0252	0.0284	0.0555
aVF	0.1747	0.9808	0.9778	0.8737	0.8210	1	0.0919	0.0291	0.0892	0.0065
PPG	0.1295	0.1127	0.0658	0.1364	0.0097	0.0919	1	0.1442	0.0078	0.0139
*S* *p* *O*_2_	0.0027	0.0280	0.0289	0.0242	0.0252	0.0291	0.1442	1	0.0624	0.4111
PTT	0.0930	0.1028	0.0708	0.1161	0.0284	0.0892	0.0078	0.0624	1	0.2262
HR	0.1062	0.0271	0.0160	0.0576	0.0555	0.0065	0.0139	0.4111	0.2262	1

**Table 4:** Ranking of the MI between the features and target

Rank		1	2	3	4	5	6	7	8	9	10
Feature		PTT	HR	PPG	aVF	III	II	*S* *p* *O*_2_	aVR	aVL	I
MI	Ps	1.000	0.933	0.647	0.216	0.290	0.208	0.171	0.164	0.159	0.106
	Pd	1.000	0.975	0.577	0.200	0.190	0.181	0.147	0.144	0.137	0.100

**Table 5:** The four feature combinations

Number	Combination	Reason
1	PTT, HR, PPG, aVF, aVR, aVL, I, II, III, *SpO*2	Use all the features
2	PTT, HR, PPG, aVF, *SpO*2	Because of a significant correlation between the fourth feature (aVF) and the fifth and sixth features (III, II) of 0.9778 and 0.9808, respectively, the fifth selected feature is *SpO*2, which is ranked seventh and is not correlated with the first four features.
3	PTT, HR, PPG, aVF	Select the 4 features with the highest dependence on the target value.
4	PTT, HR, PPG	Select the 3 features with the highest dependence on the target value.

### Comparative analysis of experimental results

In this paper, the SVR model was evaluated in terms of six evaluation indexes, and the prediction performance was analysed and compared with that of other predictive models, namely, LinearR and BP. The experimental results show that the SVR model has significant advantages over the other models. The experimental results are shown in Table 6, where Pd and Ps represent the Pd and Ps predictions.

**Table 6:** Comparison of the blood pressure predictions of each model

Index		SVR		LinearR		BP	
		Ps	Pd	Ps	Pd	Ps	Pd
**Accuracy**(%)	±3	96.43	97.14	17.43	34.00	30.86	54.00
	±5	97.71	98.43	30.71	51.14	45.00	73.14
	±7	98.43	99.00	40.57	69.43	56.86	84.00
	±10	99.00	99.43	57.14	87.14	69.43	96.43
**Pass rate**(%)	≤0.03	96.57	96.00	22.57	22.57	36.71	36.71
	≤0.05	98.00	97.14	38.29	35.71	51.86	56.57
	≤0.07	99.00	97.86	52.14	47.29	65.14	69.43
	≤0.10	99.71	99.00	65.29	63.57	75.71	82.86
**MAPE(%)**		0.3173	0.4875	8.6916	8.5605	6.6690	5.8319
**MAE**		0.4135	0.3374	11.1090	5.5047	8.4562	3.7260
**R** ^**2**^		0.9835	0.9665	0.0896	0.0546	0.3857	0.5025
**Spearman**		0.9917	0.9911	0.3341	0.2696	0.5900	0.7182

±3, ±5, ±7, ±10 and ≤0.03, ≤0.05, ≤0.07, ≤0.1 indicate the difference in error range and relative error range between the actual and predicted values

**Fig. 9:**
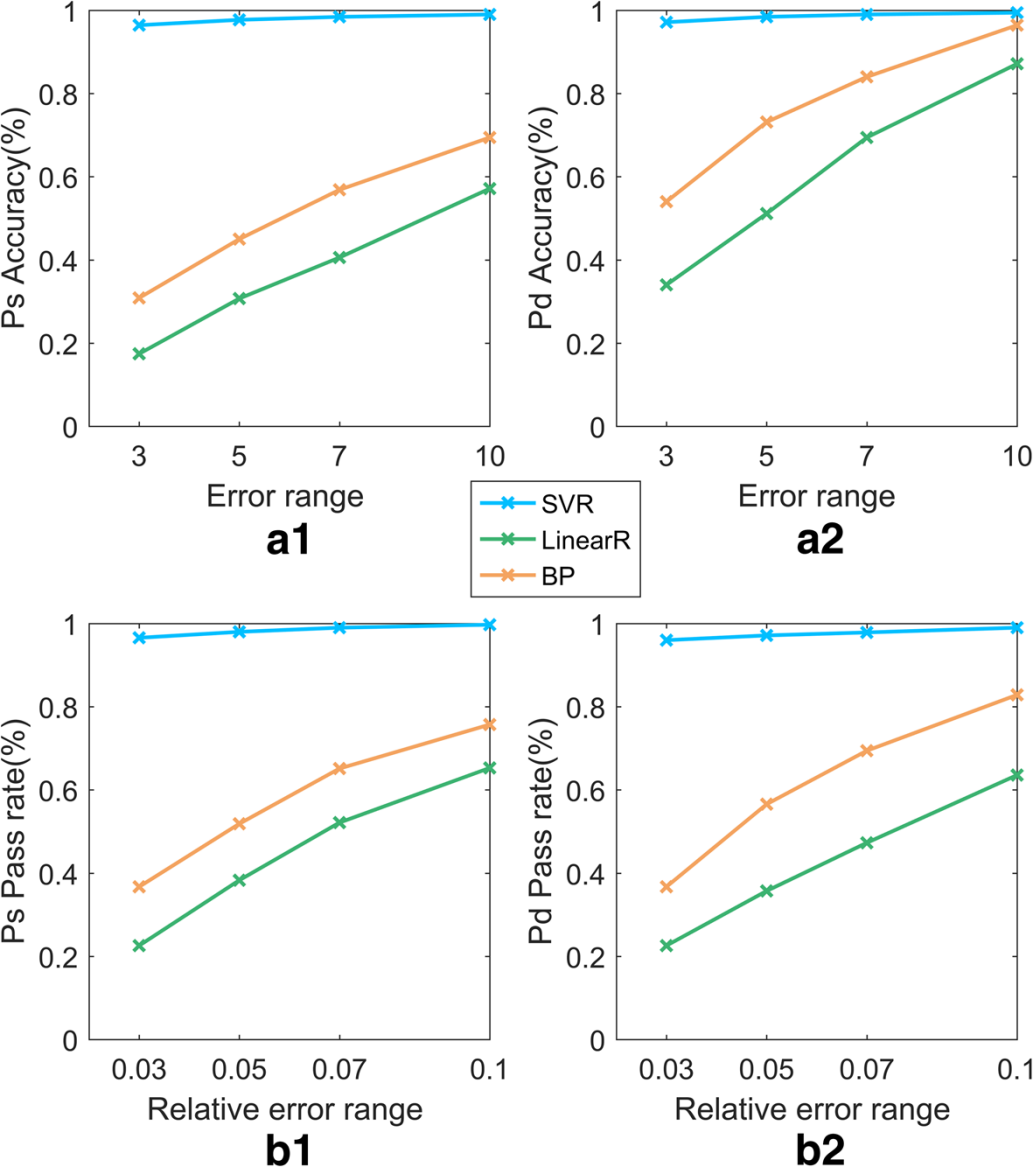
Accuracy and pass rate Comparison of 4 models (SVR, BP, LinearR). In Fig. 9, (a1) represents the accuracy for the models of Ps, whereas (a2) represents that of the models of Pd. (b1) represents the pass rate for models of Ps, whereas (b2) represents that of the models of Pd. The blue, green, orange respectively, represent the SVR, LineaR, BP

Table 6 shows that the SVR model reaches a Ps prediction accuracy greater than 96% in all four error ranges (±3 mmHg, ±5 mmHg, ±7 mmHg, ±10 mmHg). As the error range is relaxed, the accuracy of the predictions increases. The accuracy of the SVR model predictions for Pd and Ps are 97.14% and 96.43% in the range (−3 mmHg, +3 mmHg), which is much higher than those of LinearR and BP. The accuracies of the models with different error ranges are shown in Fig. 9.

Table 6 also shows the pass rate of the SVR model for three different relative error requirements (≤0.03, ≤0.05, ≤0.07, ≤0.1). The results are illustrated in Fig. 9, which shows that the prediction accuracy of the models of Ps and Pd increases as the relative error range increases. Similarly, in the minimum relative error range (≤0.03), the accuracy of the SVR model is 96.00% and 96.57% for Pd and Ps, respectively, significantly higher than the accuracies of the other models.

In addition, this paper evaluates each model in terms of MAPE, MAE, *R*^2^ and Spearman’s correlation. As a general guideline, an MAPE less than 10% indicates high prediction accuracy. Table 6 shows that the MAPE values of the Pd and Ps predicted by the SVR model are 0.4875% and 0.3173%, respectively, which are far better than 10%, indicating very good prediction performance. Furthermore, the MAE is 0.3374 and 0.4135 for Pd and Ps predictions obtained by SVR, far better than those obtained by LinearR and BP. Similarly, the *R*^2^ values are 0.9665 and 0.9835, and Spearman’s coefficients are 0.9911 and 0.9917 (the corresponding P-value are approximately 0, i.e., less than the conventional significance level of 5% (*P*<0.05)) for Pd and Ps, respectively. These values are better than those of the LinearR and BP models. From an inspection of the above evaluation indexes, it can be concluded that the values predicted by the SVR model are close to the real values. The *R*^2^ shows that the SVR model describes a large degree of the variation in blood pressure. The SVR model demonstrates its advantages compared with the other models.

## Discussion

The prediction results of the SVR, LinearR and BP models are evaluated in terms of accuracy, relative error, MAPE, MAE, *R*^2^ and Spearman’s rank correlation coefficient. The SVR model produces better predictions than the LinearR and BP models. However, to maximize the accuracy, more data are required, which inevitably increases the training time of the model and the difficulty of optimizing parameters C and gamma. In addition, the data were collected from healthy people. The efficacy of the SVR model for predicting blood pressure in the elderly, predicting abnormal blood pressure or predicting the blood pressure for different ethnic groups has yet to be verified.

There is a close relationshape between selected features (PTT, HR, PPG) in our paper and blood pressure. The blood pressure estimation approach of using PTT has been extensively studied over the past 15 years [38–43]. In recent years, blood pressure measurement with PPG has shown a lot of promise. Xing and Sun [44] provided a theoretical explanation of PPG waveforms predicting blood pressure. In addition, some studies are exploring the relationship between blood pressure and other physiological indicators, such as HR. Reule and Drawz [45] reviewed the relationship between HR and peripheral and central blood pressure. Therefore, it can be said that the use of these features to predict blood pressure is theoretically supported and feasible.

With the popularity of smart devices, it has become easier to collect various human physiological data, which provides an opportunity for multivariate analysis. Compared with the univariate prediction of blood pressure, it starts from multiple influencing factors and considers more comprehensively, so as to achieve more accurate prediction of blood pressure. In addition, the conclusions obtained from the multivariate model prediction can in turn guide further medical research and provide possible research directions.

## Conclusions

The main contribution of this paper is the use of the SVR algorithm of machine learning to investigate the implicit association between human physiological index data and blood pressure measurements collected by medical devices to obtain an efficient and accurate prediction model for human blood pressure. The SVR model proposed in this paper has achieved 98.43% and 97.71% accuracy for Pd and Ps prediction, respectively, within the American ANSI/AAMI SP10-1992 standard specified error range (±5 mmHg). In the relative error range of 5%, the prediction accuracies of Pd and Ps are 97.14% and 98.00%. The MAPEs for Pd and Ps are 0.4875% and 0.3173%, respectively, well below the generally accepted 10% standard. The MAEs for Pd and Ps are 0.3374 and 0.4135, respectively. The *R*^2^ for Pd and Ps are 0.9911 and 0.9917, respectively, which are both close to 1. We also compared the SVR model results with those obtained by a LinearR model and a neural network model: SVR achieved significantly better prediction performance.

Future research will attempt to use more physiological indicators, such as respiratory rate, body temperature, age, weight, and sleep, to construct a prediction model to discover the relationship between blood pressure diseases and other diseases and to improve and enrich current health care provisions.

## Abbreviations

BMI: Body mass index
BP: Back propagation neural network
Case GE ECG system: a 6-lead ECG
CM400: A patient monitor
ECG: Electrocardiograph
EIMO: A cuffless blood pressure measurement device
HC: Hip circumference
HR: Heart rate
LinearR: Linear regression
MAE: Mean absolute error
MAPE: Mean absolute percentage error
MI: Mutual information
Pd: Diastolic blood pressure
PPG: Photoplethysmography
Ps: Systolic blood pressure
PTT: Pulse wave transmission time
SD: Standard deviation
*S**p**O*2: Blood oxygen saturation level
SunTech Tango: criterion system
*R*^2^: R-squared coefficient of determination
RBF: Radial basis function
SVR: Support vector machine regression
WC: Waist circumference
WHR: Waist–hip ratio
